# Burden of typhoid and paratyphoid fever in India

**DOI:** 10.1056/NEJMoa2209449

**Published:** 2023-04-20

**Authors:** Jacob John, Ashish Bavdekar, Temsunaro Rongsen-Chandola, Shanta Dutta, Madhu Gupta, Suman Kanungo, Bireshwar Sinha, Manikandan Srinivasan, Ankita Shrivastava, Adarsh Bansal, Ashita Singh, Roshine M. Koshy, Dasharatha R. Jinka, Mathew S. Thomas, Anna P. Alexander, Shajin Thankaraj, Sheena E. Ebenezer, Arun S. Karthikeyan, Dilesh Kumar, K. N Swathi, Reshma Raju, Nikhil Sahai, Balaji Veeraraghavan, Manoj V. Murhekar, Venkata R. Mohan, Sindhu K. Natarajan, Karthikeyan Ramanujam, Prasanna Samuel, Nathan C. Lo, Jason Andrews, Nicholas C. Grassly, Gagandeep Kang, NSSEFI team

**Affiliations:** 1 Christian Medical College, Vellore, Tamil Nadu, India.; 2 KEM Hospital & Research Centre, Pune, Maharashtra, India.; 3 Centre for Health Research & Development-Society for Applied Studies, New Delhi, India.; 4 ICMR-National Institute of Cholera & Enteric Diseases, Kolkata, West Bengal, India.; 5 Post Graduate Institute of Medical Education & Research, Chandigarh, India.; 6 Chinchpada Christian Hospital, Nandurbar, Maharashtra, India.; 7 Makunda Christian Leprosy & General Hospital, Karimganj, Assam, India.; 8 Rural Development Trust Hospital, Bathallapali, Andhra Pradesh; 9 Duncan Hospital, Raxaul, Bihar, India.; 10 Lady Willingdon Hospital, Manali, Himachal Pradesh, India.; 11 ICMR-National Institute of Epidemiology, Chennai, Tamil Nadu, India.; 12 University of California, San Francisco, USA.; 13 Stanford University School of Medicine, Stanford, USA.; 14 Imperial College London, London, United Kingdom.

## Abstract

**Background:**

In 2017, over half the global burden of typhoid fever was projected to have occurred in India. In the absence of contemporary population-based data, it is unclear whether declining trends of hospitalization for typhoid in India reflect increased antibiotic treatment or a true reduction in infection.

**Methods:**

We conducted weekly surveillance for acute febrile illness and measured the incidence of blood culture-confirmed typhoid fever in a prospective cohort of children 6 months to 14 years old at three urban and one rural site in India between 2017 and 2020. At an additional urban and five rural sites, we combined blood culture testing of hospitalized patients with fever with health care utilization surveys to estimate incidence in the community.

**Results:**

24,062 children were enrolled across four cohorts, contributing 46,959 child years of observation (CYO). 299 culture-confirmed typhoid cases were recorded, with incidence per 100,000 CYO of between 576 and 1173 in urban sites, and 35 in rural Pune. The estimated incidence of typhoid fever from hospital surveillance ranged between 12 and 1622 per 100,000 CYO in children 6 months to 15 years, and between 108 and 970 per 100,000 person-years among those above 15 years, although there was more uncertainty in these estimates. *S*. paratyphi was isolated from 33 children, overall incidence of 68 per 100,000 CYO after adjusting for age

**Conclusions:**

The incidence of typhoid fever in urban India remains high.

An estimated 14.3 million cases of enteric fever caused by *Salmonella enterica* serovar typhi and paratyphi (*S*. typhi and *S*. paratyphi) occurred globally in 2017, with *S*. typhi responsible for 11 million cases of typhoid fever and 120,000 deaths.^1^ Over half the deaths were in children under 15 years. Since 2008, the World Health Organization (WHO) has recommended typhoid vaccination, but the uptake of available vaccines was limited as they could not be used in young children.^2^ Vi polysaccharide-based typhoid conjugate vaccines (TCV) that are now available were recommended again in 2018 by WHO, prioritising countries with the highest burden.^3–6^

Despite substantial burden, there is limited systematic surveillance and marked variation in incidence within and between countries.^7^ Further, true incidence may be masked by widespread use of antibiotics.^8^ Resistance to azithromycin, widely used following emergence of fluoroquinolone resistance, and resistance to third generation cephalosporins are emerging concerns.^9,10^

In India, with over half the estimated global burden of typhoid, public vaccination remains unimplemented in part related to the lack of contemporary disease burden data. We established the Surveillance for Enteric Fever in India (SEFI) to address evidence gaps identified by the National Technical Advisory Group on Immunization and report results from four community cohorts (Tier 1 surveillance), and six Tier 2 sites that combined hospital surveillance with local healthcare utilization surveys (Figure 1).

## Methods

### Tier 1 surveillance – community based active surveillance

**Figure 1:**
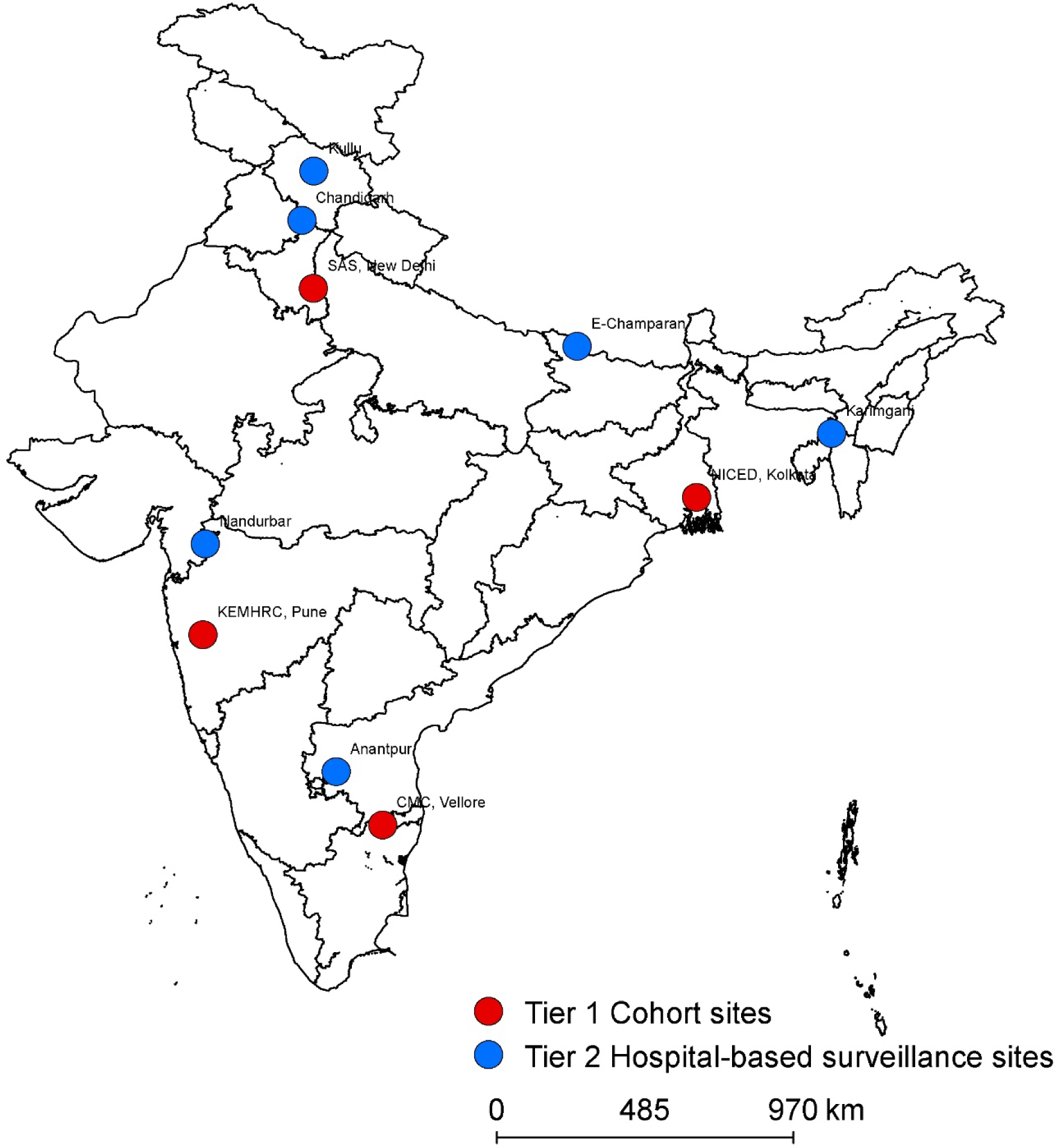
Location of study sites in India. Cohort and hospital-based (‘hybrid’) surveillance sites are shown.

For four prospective, closed cohorts in India (Delhi, Kolkata and Vellore (urban) and Pune (rural); Figure 1), we enrolled ~6,000 eligible children between 6 months and 13 years of age at each site between October 2017 to February 2018, and followed each child for 24 months or until their 15^th^ birthday. These study sites were chosen for broad urban/rural and geographic representation across the country, while ensuring their ability to perform high-quality community-based research. The protocol and informed consent forms were approved by the Christian Medical College Institutional Review Board and by ethics committees at each of the sites. All children in the defined catchment area were eligible and written informed consent was obtained from the primary caregiver. A detailed study protocol, including sample size calculations, has been published (and at nejm.org) and the study registered (Clinical Trial Registry of India CTRI/2017/09/009719).^11^

Weekly surveillance for fever was conducted either by telephonic interviews or a home-visit, with one mandatory home-visit each month. A digital thermometer and fever diary were provided to report any acute febrile illness (AFI). An initial report of fever triggered a home, study clinic or hospital visit to assess the child. Daily visits continued until the resolution of fever, defined by three consecutive fever-free days. Physician-assigned diagnosis, clinical investigations, and treatment, including antibiotics, were recorded.

For children with potential enteric fever (PEF), defined as fever for three or more consecutive days, an age-appropriate blood volume was inoculated into a BacT/ALERT^®^ or BACTEC^®^ bottle. Prior antibiotics were not considered a contraindication. *S*. typhi and *S*. paratyphi were isolated based on standard laboratory methods,^11,12^ and antimicrobial susceptibility determined following Clinical and Laboratory Standards Institute guidelines.^13^

The incidence rates of AFI, PEF, typhoid and paratyphoid fever were calculated per child year of observation (CYO) using survival analysis with interval censoring of periods when valid recall was unavailable. The overall incidence was adjusted for age structure at each site. We investigated the association of typhoid incidence with baseline household characteristics and location using Andersen-Gill’s proportional hazards model.^14^ We estimated incidence with and without adjustment for the sensitivity of blood culture (60%).^8^ Details of the statistical analysis are provided in Supplementary Appendix.

### Tier 2 surveillance – Hospital based surveillance combined with health utilization surveys

At six hospitals in additional locations representing different geographic and risk settings, study physicians screened hospitalized patients over 6 months old for fever and following consent, performed blood cultures (BACTEC^®^) between February 2018 and March 2020 (Supplementary Appendix; Figure 1). These study sites were chosen to represent geographical, urban/rural, and risk settings across India. These sites were often in regions with poor health and research infrastructure from which data are rarely obtained and the selected hospitals provided care for a significant proportion of the studied population. Further description of each site is available in the Supplementary Appendix. An independent agency conducted Healthcare Utilization Surveys in each hospital’s catchment to estimate the proportion of the population that used the surveillance hospital(s) for fever related hospitalization.^14^

The estimated incidence of hospitalized typhoid and paratyphoid fever among children (6 months to 14 years) and older individuals (15 years plus) in the catchment population was calculated by dividing the number of culture-confirmed cases by population size, adjusting for the age-specific proportion of febrile illnesses seen at the surveillance hospital from the Healthcare Utilization Surveys (Figure S1). The catchment population was defined as the contiguous area from which 80% of hospitalizations in the previous 2 years were recorded. Incidence was adjusted further for the proportion of eligible episodes where blood cultures were not done because of non-consent and for the sensitivity of blood culture. To estimate the incidence of typhoid of all clinical severity, we divided the estimates of hospitalized typhoid fever by the proportion of typhoid fevers that required hospitalization in Tier 1 surveillance.^12^ This adjustment approach for hybrid surveillance studies has been previously done for enteric fever.^15–17^ To account for uncertainty in the adjusted estimates, we used Monte Carlo simulation that samples a range of values for each multiplier used from the Healthcare Utilization Surveys (using a beta distribution) and report the 95% uncertainty interval (Tables S7, S8).^18^ We performed sensitivity analyses on the blood culture sensitivity when accounting for prior antibiotic exposure (See Supplementary Appendix).

## Results

### Tier 1 surveillance cohort characteristics

**Figure 2:**
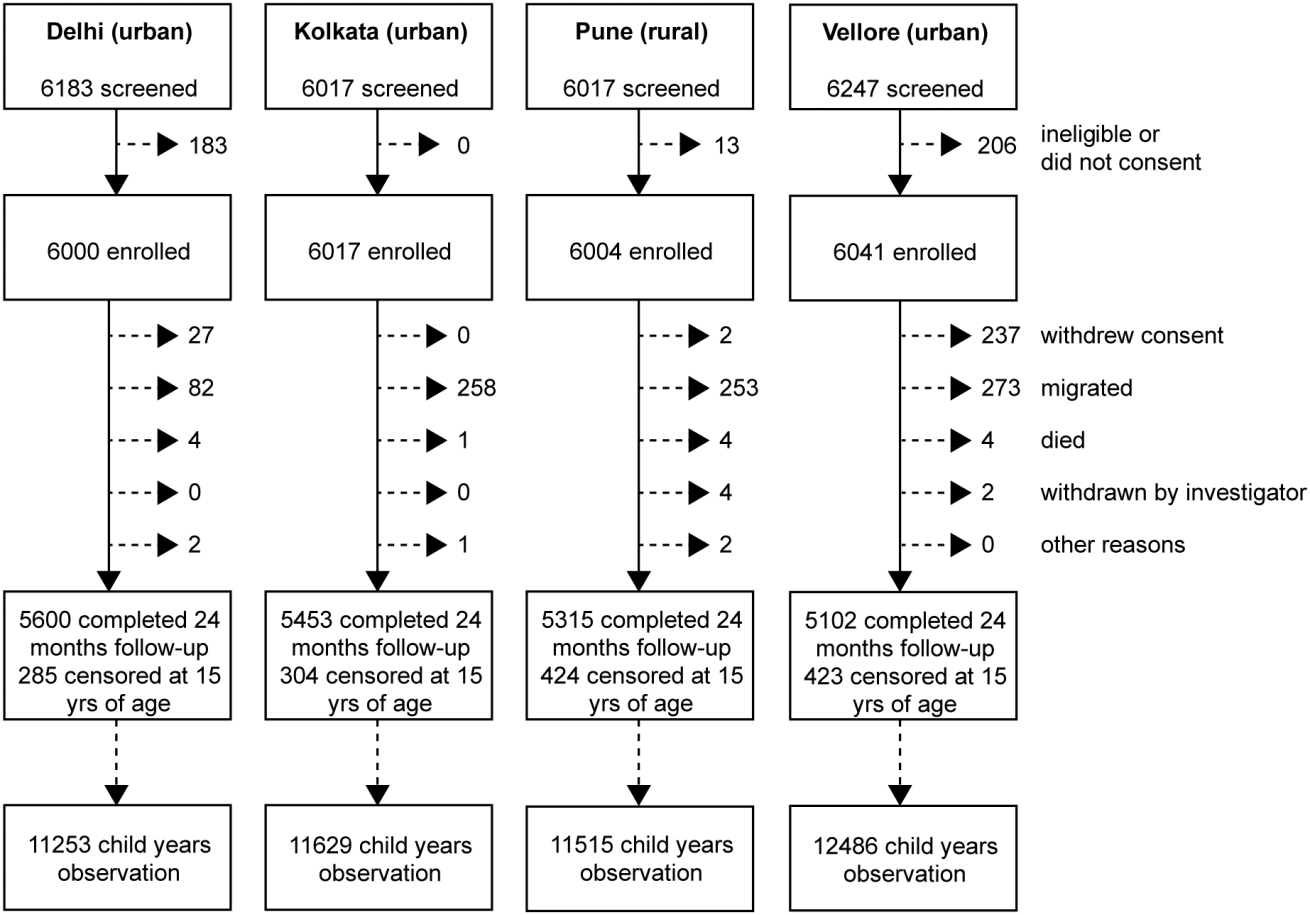
Flow diagram indicating the numbers of children enrolled at each site, completion of 24 months follow-up and number of child years of observation. Reasons for loss to follow-up are shown on the right.

A total of 24,062 children were enrolled at four sites, with 21,470 (89.2%) completing 24 months of follow-up, 1,436 (6.0%) censored at 15 years, and 1,156 (4.8%) lost to follow-up or withdrawing consent (Figure 2). Study populations were three urban poor, densely populated city catchments and a rural agrarian community across several villages (Table S1). Drinking water sources and toilet facilities varied significantly between sites (p-values <0.001); 603 (4.3%) households had access only to unimproved water,^19^ 3189 (22.6%) used bottled water (mainly in Pune) and the remainder had access to piped water or a public tap. Water treatment was inadequate for 10,425 (73.7%) children. The majority (84.2%) of households had access to toilets, although 37.2% shared toilets with other families.

### Incidence of AFI and PEF in Tier 1 cohorts

We recorded 76,027 AFIs during 46,959 CYO across the four cohorts, with the highest incidence among children aged between 6 months and 4 years (2.45 episodes per CYO; Table S2). Overall incidence among children aged 6 months to 14 years old was 1.73 episodes per CYO. PEF criteria were met in 20,911 (27.5%) fevers (range 24.2–29.2% among sites), of whom 70.1% (range 67.0–75.8%) remained febrile at investigation and were eligible for a blood culture. Blood cultures were done for 86.6% (range 79.1–93.6%) of eligible PEFs and the remainder were not done because of refusal by the caregiver (7.3%) or other reasons (6.1%), mainly being outside the study area. The pre-specified blood volume was obtained for 91.0% of cultures. Antibiotics were used in 67.7% of PEFs, 38.8% before blood culture (range 22.4–64.8% across sites). Blood cultures were performed on day 4 of fever in 67.6%, on day 5 in 18.8% and on day 6 to 16 for the remainder (13.6%). The clinical diagnoses of these episodes are listed in Table S3.

### Incidence of culture-confirmed typhoid or paratyphoid fever in Tier 1 cohorts

**Figure 3:**
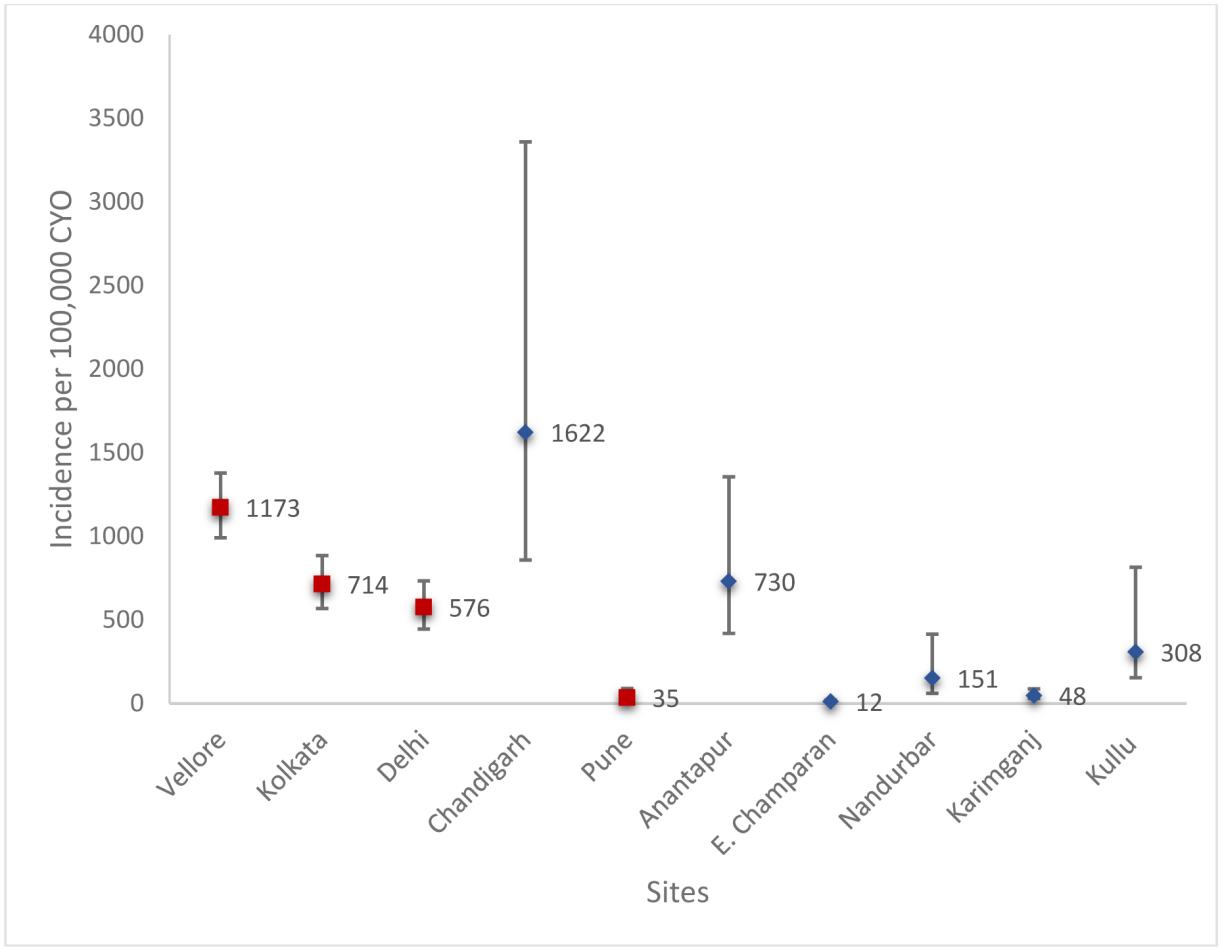
Adjusted incidence of typhoid fever in children aged 6 months to 14 years from urban and rural cohorts and hospital-based surveillance. Cohort incidence estimates (red points) without adjustments. Hospital-based incidence estimates (blue points) are adjusted for health care seeking in the corresponding age-group, missed blood collection due to non-consent, the proportion of typhoid cases that require hospitalization and blood culture sensitivity. Error bars show 95% uncertainty intervals based on the Poisson distribution or Monte Carlo simulation respectively.

There were 299 culture confirmed cases of typhoid, ranging from just 4 in Pune to 146 in Vellore (Table 1). Overall incidence among children aged 6 months to 14 years per 100,000 CYO was 576 (95% confidence interval (CI): 445–734) in Delhi, 714 (568–885) in Kolkata, 35 (9–89) in Pune and 1173 (991–1379) in Vellore, after adjusting for the age-distribution of the underlying population (Figure 3). Incidence was highest among children 5–9 years old in Vellore, Kolkata and Pune sites and 10–14 years old in Delhi (Table 1; Figure S2). *S*. paratyphi was isolated from 33 children, giving an overall incidence of 68 (95% CI: 47–96) per 100,000 CYO after adjusting for age.

The incidence of typhoid varied over time but did not show seasonality or association with wetter monsoon months (hazard ratio (HR) 0.95 (95% CI: 0.69–1.32), p=0.77; Figure S2) at any site. In the final multivariable model, the risk of typhoid was greater for children from households of greater than average size, with fewer assets and without a sanitary toilet (Table S4). Typhoid incidence was 648 (95% CI, 568 – 739) per 100,000 PYO in those without access to safe water compared to 611 (489 – 763) per 100,000 PYO in those with access to safe water. Incidence of typhoid was lower among vaccinated children, but few children were vaccinated, and this difference was not significant (HR=0.60 (95% CI: 0.28–1.27), p=0.18).

### Clinical characteristics of typhoid cases in Tier 1 cohorts

The median duration of culture-confirmed typhoid fever was 9 days (interquartile range 7–11 days) and the median highest temperature was 102.8 °F (Table S5). Other than fever, common symptoms were cough (145/299), nausea or vomiting (139/299), abdominal pain (137/299) and headache (115/299) (Table S5; Figure S3). Clinical characteristics of paratyphoid were similar to typhoid. No children with typhoid or paratyphoid died although 46 (15%) and 7 (21%), respectively, were hospitalized.

### Antibiotic use and sensitivity among confirmed typhoid fever cases in Tier 1 cohorts

Nearly all children with confirmed typhoid received antibiotics (296/299), mainly azithromycin (230/299) followed by cephalosporins (145/299) (Table S5). Administration of multiple antibiotics was common (160/299). Nearly all (289/294) were non-susceptible to ciprofloxacin.

### Tier 2 surveillance hospital admissions

15,736 (79%) of 20,022 febrile admissions between February 27, 2018, and March 31, 2020, were recruited at the six hospitals, with 8253 (53%) males, and 6120 (39%) <15 years of age (Table S6).

Blood cultures were obtained from 13,264 (84%) participants, with 8.9 ml mean blood volume in adults and 3.5 ml in children. The most common pathogen was *S*. typhi (n=221; 1.7%), followed by *Staphylococcus* species (n=197; 1.5%) and *Escherichia coli* (n=178; 1.3%). *S*. paratyphi A was recovered from 54 samples (0.4%), with one *S*. paratyphi C. Of these, 185 *S*. typhi and 52 *S*. paratyphi A were from patients within the catchment population. In 7.8%, blood culture was performed after antibiotic initiation.

### Incidence of culture-confirmed typhoid and paratyphoid fever at Tier 2 surveillance sites

After adjusting for surveillance coverage, study compliance, severity and blood culture sensitivity, the adjusted incidence of typhoid fever in children per 100,000 CYO ranged between 12 (95% Uncertainty Interval (UI): 7–20) in East Champaran and 1622 (858–3359) in Chandigarh (Table 2, Table S7). The incidence of paratyphoid per 100,000 CYO ranged between 0 at multiple rural sites and 696 (368–1439) at urban Chandigarh site (Table S8). Among those over 15 years, the adjusted incidence of typhoid fever per 100,000 person-years varied between 108 (69–177) in Karimganj and 970 (683–1420) in Chandigarh (Table S7), and for paratyphoid fever between 8 (5–13) in Karimganj and 416 (305–607) in Chandigarh (Table S8). Sensitivity analyses that adjust for blood culture sensitivity in those with prior antibiotic exposure is presented in Tables S9–S10.

The median durations of fever and hospitalization in those with typhoid fever were nine and five days, respectively. The median highest temperature recorded was 102.4°F. The median age of those hospitalized with typhoid fever was 19.4 years (IQR, 10.1 to 24.7 years), with 54.3% of cases aged between 15 and 30 years. Of the 221 patients with typhoid fever, 202 recovered without complications, 14 were referred to higher centres, four left against medical advice, and one died.

## Discussion

During two years of active surveillance, the incidence of blood culture-confirmed typhoid in 3 urban Indian cohorts of children exceeds published thresholds above which routine vaccination with TCV becomes highly cost-effective.^20,21^ It is substantially higher than burden estimates based on mathematical models fit to hospital-reported cases^22^ and is comparable with the reported incidence from cohort studies conducted between 1995 and 2006.^23–25^ Estimated incidence in children is even higher when accounting for the low sensitivity of blood culture with the adjusted incidence ranging between 960 (742–1223) in Delhi to 1995 (1652–2298) at Vellore. These estimates are also consistent with the estimates in children in urban Chandigarh from the Tier 2 hospital-based surveillance.

The high incidence of typhoid in urban India suggests that declines in reported cases from hospitals have been driven by changes in healthcare-seeking behaviour and widespread antibiotic treatment, rather than a reduction in transmission. Antibiotics are commonly available without a prescription and the number of defined daily doses used in India has increased from 3.2 to 6.5 billion per year between 2000 and 2015.^26^

The apparent masking of typhoid disease by widespread use of effective antibiotics highlights the major risk posed by emerging strains of extensively drug resistant *S*. typhi, strengthening the argument for TCV introduction.^10^ Although disease incidence peaks among children aged 5–9 years, there is a significant burden from one year of age. Routine immunization with a single dose of TCV at 9 months of age following WHO recommendations would offer early protection, with a one-time catch-up immunization of children up to 15 years of age providing protection in a larger vulnerable age range.^5^

The estimated incidence of typhoid fever in children in rural areas was lower than in urban areas. Although urban vaccination alone may reduce incidence, rural areas continue to have moderate levels of disease, and urban migration of younger people will threaten gains achieved through an urban-only vaccination strategy. Over half the hospitalized typhoid fever episodes in rural settings were among individuals over 15 years of age indicating a substantial burden in adults that may reflect lower exposure during childhood and consequent vulnerability if they migrate to higher risk settings.

The geographic heterogeneity adds complexity for immunization programs and is also an important issue elsewhere, with many rural studies in Africa and Asia reporting low incidence of *S*. typhi,^27,28^ but other rural or peri-urban sites recording relatively high incidence.^29^ However, the uncertainty of typhoid estimates in rural areas is larger given that the majority of rural data came from the Tier 2 study.

The most administered antibiotics for PEF were azithromycin or cephalosporins (cefixime or ceftriaxone). The predominant genotype of *S*. typhi circulating in India (4.3.1 also called H58) remains susceptible to these antibiotics and all isolates were susceptible to ceftriaxone. Extensively drug resistant (XDR) *S*. typhi strains with resistance to third generation cephalosporins reported from Pakistan have not yet been isolated in India.^10^ However, six *S*. typhi isolates and eight *S*. paratyphi A isolates showed evidence of resistance to azithromycin. This is consistent with the emergence of azithromycin resistance elsewhere.^9^ The spread of *S*. typhi strains resistant to azithromycin and other first-line antibiotics would change typhoid treatment from oral antibiotics to needing parenteral antibiotics in an inpatient setting, risking higher rates of mortality and putting a further strain on health systems. The burden of paratyphoid was variable and lower in Tier 1 than at Tier 2 sites. The variable incidence and the observed antimicrobial resistance in *S*. paratyphi are of particular importance as typhoid conjugate vaccines do not protect against *S*. paratyphi

Our studies had several limitations. In the active surveillance cohorts, early treatment with antibiotics may have resulted in the resolution of fever in some children meaning they were ineligible for blood culture, potentially contributing to an underestimate of the true incidence. We aimed to minimise this bias through weekly fever surveillance and early referral for blood culture. Nonetheless, 30% of PEFs were ineligible for blood culture because of defervescence, of whom 28% had received antibiotics for at least 48 hours. Prior analyses from this study on the relationship of antibiotics and culture positivity did not yield a relationship.^30^

We were also unable to conduct a blood culture in 13% of eligible PEFs and in 9% we did not obtain sufficient blood volumes, meaning we may have missed some episodes. The 10 study sites (4 in Tier 1 and 6 in Tier 2) were not randomly selected and thus not entirely representative of India, and therefore we do not provide a national incidence estimate. These sites were chosen to broadly reflect the geographic, urban/rural, and risk settings available in India, but achieving full representativeness was not possible given the size of the country and the intensive resources needed to conduct such studies.

The Tier 2 estimates on enteric fever are more uncertain than Tier 1. Estimates of typhoid incidence from hospital-based surveillance depend on accurate multipliers used to account for healthcare seeking, disease severity, blood culture administration and sensitivity which all retain significant individual uncertainty. The overall magnitude of the product of these multipliers leads to considerable uncertainty in the estimation of typhoid and paratyphoid fever incidence from hospital admission data. To address this limitation, we derived uncertainty intervals using Monte Carlo simulation and based our estimates of healthcare utilization on surveys from 5000 households at each site, which demonstrate appropriately wide intervals suggesting uncertainty.^31^ This hybrid surveillance methodology has been applied in infectious diseases surveillance across multiple pathogens^15–17^ Finally, the Tier 2 surveillance did not perform sample size calculations to inform study design.

In summary, despite improvements in water and sanitation and declining trends reported from hospitals, the burden of typhoid in urban India remains high, particularly among the poorest households. TCV introduction, in parallel with continued emphasis on safer water, sanitation and food, offers potential for reduction of an under-estimated and often overlooked infection disease in a country that carries the highest burden of typhoid.

## Supplementary Material

supplement

## Figures and Tables

**Table 1:** Incidence of culture confirmed typhoid and paratyphoid fever across the four Tier 1 cohorts

	Vellore	Kolkata	Delhi	Pune	Overall
Febrile illness	23 548	17 741	14 439	20 299	76 027
Potential enteric fever	6878	4285	4198	5550	20911
Blood cultures reported	4156	2293	2533	3701	12 683
*S*. typhi positive (%)	146 (3.5)	81 (3.5)	68 (2.7)	4 (0.1)	299 (2.4)
*S*. paratyphi positive (%)	1 (<0.1)	13 (0.6)	12 (0.5)	7 (0.2)	33 (0.3)
Number of typhoid fevers (incidence rate per 100 000 CYO, 95% CI)					
0.5–4 years	30 (974, 681–1392)	20 (662, 427–1027)	13 (459, 267–791)	0 (0, 0–89)	63 (536, 419–687)
5–9 years	68 (1341, 1057–1700)	39 (958, 700–1311)	29 (618, 429–889)	3 (71, 23–220)	139 (770, 652–909)
10–14 years	48 (1096, 826–1454)	22 (485, 319–736)	26 (693, 472–1017)	1 (22, 3–158)	97 (566, 464–690)
Total*	146 (1173, 991–1379)	81 (714, 568–885)	68 (576, 445–734)	4 (35, 9–89)	299 (637, 567–713)
Number of paratyphoid fevers (incidence rate per 100 000 CYO, 95% CI)					
0.5–4 years	0 (0, 0–82)	3 (99, 32–308)	2 (71, 18–282)	2 (71, 18–284)	7 (60, 28–125)
5–9 years	0 (0, 0–50)	7 (172, 82–361)	2 (43, 11–170)	2 (47, 12–189)	11 (61, 34–110)
10–14 years	1 (23, 3–162)	3 (66, 21–205)	8 (213, 107–426)	3 (67, 22–208)	15 (87, 53–145)
Total*	1 (8, 1–44)	13 (112, 60–191)	12 (98, 49–174)	7 (61, 24– 125)	33 (68, 47–96)

* Adjusted to match the underlying age distribution

**Table 2:** Estimated incidence of typhoid and paratyphoid fever in hospital-based Tier 2 surveillance

Site	Setting	Geographic region	Number of hospitalized typhoid fevers	Person Years of Observation (CYO)	Estimated incidence of typhoid fever of any severity per 100 000 CYO (95% UI)	Number of hospitalized paratyphoid fevers	Estimated incidence of paratyphoid fever of any severity per 100 000 CYO (95% UI)
Less than 15 years							
Chandigarh	Urban	Northern	21	71595	1622 (858–3359)	9	696 (368 – 1439)
Anantapur	Rural	South	19	194244	730 (419–1356)	3	115 (66 – 214)
E. Champaran	Rural	Northern Gangetic	1	392098	12 (7 – 21)	0	-
Nandurbar	Rural	Western, remote	2	129095	151 (61–415)	0	-
Karimganj	Rural	North-eastern	6	260043	48 (31 – 88)	0	-
Kullu	Rural	Himalayan	5	51210	308 (155 – 815)	0	-
15 years and older							
Chandigarh	Urban	Northern	71	193571	970 (683 – 1420)	32	437 (308–641)
Anantapur	Rural	South	8	776975	119 (73 – 202)	0	-
E. Champaran	Rural	Northern Gangetic	15	667627	130 (81 – 218)	4	34 (22–58)
Nandurbar	Rural	Western, remote	7	485642	174 (98 – 317)	1	25 (14–45)
Karimganj	Rural	North-eastern	13	504789	108 (70 – 177)	1	8 (5–14)
Kullu	Rural	Himalayan	17	192650	275 (172 – 469)	2	32 (20–55)

Further description of the characteristics of the Tier 2 surveillance sites is available in the Supplemental Appendix
