# Supplementary material for: Burden of typhoid and paratyphoid fever in India
Source: N Engl J Med. Author manuscript; Available in PMC 2023 Apr 20. (PMC10116367; doi:10.1056/NEJMoa2209449)
Supplement: supplement [file NIHMS1868189-supplement-supplement.pdf]

Supplementary Appendix for

Burden of typhoid and paratyphoid fever in India

Table of contents

NSSEFI Team 2
Supplementary Methods 3
Supplementary Tables 11
Supplementary Methods References 22

NSSEFI Team

<u>Tier 1 Cohort surveillance sites</u>

Christian Medical College, Vellore, Tamil Nadu, India

Jacob John, M.D., Ph.D.

Manikandan Srinivasan, M.D.

Prabakhar D. Moses, M.D., FRCP

Winsley Rose, M.D.

Priscilla Rupali, M.D. Ph.D.

Kulandaipalayam Natarajan Sindhu, M.D.

KEM hospital Research centre, Pune, Maharashtra, India

Ashish Bavdekar, MBBS, D.C.H., D.N.B.

Ankita Shrivastava, BAMS, M.P.H.

Sonali Sanghavi, M.D., Ph.D.

Sanjay Juvekar, Ph.D.

ICMR- National Institute of Cholera & Enteric Diseases, Kolkata, West Bengal, India

Shanta Dutta, M.D., Ph.D.

Suman Kanungo, M.B.B.S Ph.D.

Jayanta Saha, M.B.B.S.

Pranab Chatterjee, M.D.

Centre for Health Research & Development-Society for Applied Studies, New Delhi, India

Temsunaro Rongsen-Chandola MBBS, Ph.D.

Bireshwar Sinha, M.D.

Nidhi Goyal, MBBS, M.Sc.

Deepak More, M.D.

Alok Arya, M. Pharm.

Ankita Dutta, M.Sc.

Chandra Mohan Kumar, M.D.

<u>Tier 2 Hospital based hybrid surveillance</u>

Post Graduate Institute of Medical Education and Research, Chandigarh, India

Madhu Gupta, M.D. Ph.D.

Adarsh Bansal, M.P.H.

Makunda Christian hospital, Assam, India

Roshine Mary Koshy, M.D.

Shajin Thankaraj, M.D., DNB

Chinchpada Christian hospital, Navapur, Maharashtra, India

Ashita Singh, M.D., M.A.

Alice Hepzibah, C.H., R.N., R.M., M.Sc.

Lady Willingdon Hospital, Manali, Himachal Pradesh, India

Anna P. Alexander, M.D.
Pradeep Zachariah, M.S.
Christina Dhas Sankhro, M.B.A,
Rural Development Trust Hospital, Anantapur, Andhra Pradesh, India
Dasaratha Ramaiah Jinka, M.D.
Raghuprakash Reddy Nayakanti, M.D.
Duncan Hospital, Raxaul, Bihar, India
Sheena Evelyn Ebenezer, M.D.
Mathew Santosh Thomas, M.D.
ICMR- National Institute of Epidemiology, Chennai, Tamil Nadu
Manoj V. Muhrekar, Ph.D.
Elangovan A, Ph.D.

Study coordination (Christian Medical College, Vellore)
Gagandeep Kang, M.D., Ph.D.
Balaji Veeraraghavan, M.D. Ph.D.
Venkata Raghava Mohan, M.D.
Arun S Karthikeyan, Ph.D.
Dilesh Kumar, M.P.H.
Swathi Krishna N, M.D.
Miriam T. George, Ph.D.
Reshma Raju, M.D.
Karthikeyan Ramanujam, M.Sc.
Santhosh Kumar G, M.Phil
Prasanna Samuel, Ph.D.
Nikhil Sahai, B.Tech
Agila K Pragasam, Ph.D.
Jobin J John, Ph.D.
External Collaborators
Nicholas C. Grassly D.Phil., Imperial College London, United Kingdom
Jason Andrews, M.D. Stanford University School of Medicine, Stanford, USA
Nathan C. Lo, M.D. Ph.D, University of California, San Francisco, USA.

Supplementary Methods

Tier 1 statistical analysis

Baseline household characteristics were compared between sites using the chi-squared test. The incidence rates of fever, PEF, and typhoid and paratyphoid fever were calculated per child year of observation (CYO) using survival analysis with interval censoring of periods where valid recall was unavailable. Repeated occurrence of events was

permitted in the analysis. Age-group specific incidence was estimated using the 'stsplit' function in STATA and the overall incidence calculated as an average across age-groups, weighted to reflect the age structure of the underlying population at each site. Confidence intervals were calculated using the quadratic approximation to the Poisson log-likelihood for the log-rate parameter. We investigated the association of typhoid incidence with baseline household characteristics and location using Andersen-Gill's proportional hazards model.[1] We built the model incorporating prespecified household (overcrowding, type of family, family size, separate kitchen, and low assets score), environmental (type of house, safe water, treatment of water, sanitary practice of child, sanitary practice of household, and sharing of toilet) and individual (gender, vaccination) level variables and selected the final model based on the Akaikie Information Criteria (AIC). The analysis was performed using STATA 14.2 (StataCorp. 2015. Stata Statistical Software: Release 14. College Station, TX: StataCorp LP.) and R programming language (R version 3.6.1).

Tier 1 notification

We did not explicitly measure the duration of time between notification and visit in the cohort study. However, since most of the weekly surveillance visits were in-person, the bulk of the notifications for cases were at the time of the first visit. When notifications were identified during the telephonic contact, visits were initiated within one day. We found that the mean interval between the onset of fever and the first visit was 1.89 days (sd 1.31days).

Tier 2 Hospital based surveillance sites

The surveillance sites were chosen to represent different geographic and risk settings such as climate and population density. We considered the site's ability to conduct research and the proportion of the catchment population accessing healthcare at the study hospital. Since all sites were required to conduct blood cultures on all febrile admissions, this necessitated the establishment of culture facilities at some facilities, with quality control procedures including proficiency testing at all facilities. All sites provided data on febrile hospitalizations during 2016 and 2017, and geographically contiguous administrative areas from which 80% of the hospitalized febrile patients came to the study facility constituted the catchment for each site.

A brief description of surveillance sites follows.

1) The Anantapur site in the southern state of Andhra Pradesh had a catchment population of 487,000 served by the 350-bed Rural Development Trust Hospital (RDT) at Bathallapali
2) The Nandurbar site in the western state of Maharashtra had a population of 311,000 with a predominantly tribal population was served by Chinchpada Christian Hospital, a 50-bed charitable hospital.

3) The East Champaran site in Bihar is one of the most backward districts of the country. This site has a catchment population of 530,000 in the Raxaul and Ramgarhwa blocks and is served by the Duncan Hospital, Raxaul, a 200-bedded surveillance facility.
4) The Karimganj site in the north-eastern state of Assam is located at the tri-junction of Assam, Tripura, and Mizoram. The Makunda Christian Leprosy and General Hospital is a 160-bed charitable facility that serves Lowairpoa and Patharkandi blocks with a population of 384,000.
5) The Kullu site in the Himalayan ranges of Himachal Pradesh, with a catchment population of 123,000, is served by the 55-bed Lady Willingdon Hospital in Manali.
6) Chandigarh, the only urban site, identified a population of 143,000 in Sectors 45 and 52 as its catchment population. The Civil Hospital at Sector 45 Chandigarh, a 50-bedded government hospital, was the primary inpatient healthcare facility for this population.

Establishment of the hospital surveillance system

Physicians recruited patients older than six months hospitalized at each study facility with presenting complaint of fever, irrespective of the duration or recorded temperature. At admission, study personnel took informed consent and collected age-appropriate blood samples for blood culture. The patients were managed using site-specific treatment protocols. The study protocol required no intervention other than blood culture and data collection. We collected sociodemographic information, history of prior treatment, clinical and laboratory data on electronic case report forms (CRF). The data was stored on a secured, cloud-based, custom-built data management system. Patients with blood-culture confirmed enteric fever were monitored daily to document temperature trends, antibiotic therapy, and complications if any.

Quality control of data and monitoring

The study protocol was harmonised by the coordination unit at the Christian Medical College, Vellore (CMCV), in consultation with the Scientific Advisory Process for Optimal Research on Typhoid (SAPORT), an advisory group established by the Bill and Melinda Gates Foundation, with participation from the World Health Organisation. The coordination unit monitored sites through frequent site visits and data validation checks. Deviations from the protocol were addressed by joint review and re-training. Laboratories participated in an external quality assurance system.

Laboratory methods

At admission, age-appropriate blood volumes (3 ml for infants; 5 ml for those between 1 and 15 years; 8 -10 ml for those older than 15 years) were collected and cultured using an automated system (BD BACTEC™ blood culture system). All the *S*. Typhi and *S*. Paratyphi isolates were reconfirmed and characterised for antimicrobial susceptibility at the central laboratory at CMCV.

Healthcare utilization survey

Two rounds of healthcare utilization surveys were conducted in 2018 and 2019 to identify the proportion of all febrile hospitalizations from the catchment population at the study hospital. A two-stage cluster sampling strategy was used for the selection of households. In the first stage, a random sample of 100 geographical clusters was selected in each site using probability proportional to size method. In the second stage, 50 households were selected from each cluster by systematic random sampling. The methods of the healthcare utilization survey are detailed elsewhere.[2]

Incidence calculation and statistical methods

Crude incidence of severe (hospitalized) enteric fever was calculated by dividing the number of culture-confirmed typhoid and paratyphoid fevers by the catchment population and adjusting for the period of surveillance. The catchment area population was estimated based on projections for the year 2019 from the 2011 census. Only culture-positive enteric fever cases at the study facility from the catchment area contributed to the numerator.

Given the many factors that affect enteric fever incidence measurement through hospital-based surveillance, we performed adjustments (Supplementary Figure S1). Since only a subset of all febrile hospitalizations occurred at the surveillance facility, we adjusted for the proportion of illness with similar presentations treated elsewhere based on the healthcare utilization survey (A2). This ranged from 0.10 in Nandurbar to 0.38 in Chandigarh and Karimganj.[2] A subset of participants admitted with fever failed to receive a blood culture either because of non-consent or operational reasons (ranging from 0-40% among sites), and they were assumed to have a risk of typhoid like those who received a blood culture at the same age and site (A3). We adjusted for the poor sensitivity of blood culture using an adjustment based on blood volume inoculated in culture. (A4). The correction for blood culture sensitivity was assumed to be 60% with an uncertainty range from 50-70%.[3] These adjustments were incorporated in a probabilistic multiplier model. A Monte Carlo approach using a beta distribution generated 1,000 randomly sampled iterations to account for uncertainty in the multiplication parameters. We used the simulation

results to obtain the median and 95% uncertainty intervals for the number of total cases of febrile hospitalizations in the catchment area (Supplementary Table S7, S8).

As an additional analysis to permit comparison with studies that estimate the incidence of enteric fever of all severity, we adjusted our estimates of severe enteric fever for the proportion of enteric fevers that require hospitalization (0.154) in cohort studies that formed the tier 1 surveillance (A1). We performed a sensitivity analysis using a lower blood culture sensitivity of 40% (varied 30-50%) for the site- and age-specific fraction of study participants who received antibiotics prior to blood culture collection. Statistical analysis was performed using STATA 15.0 and R 3.6.1

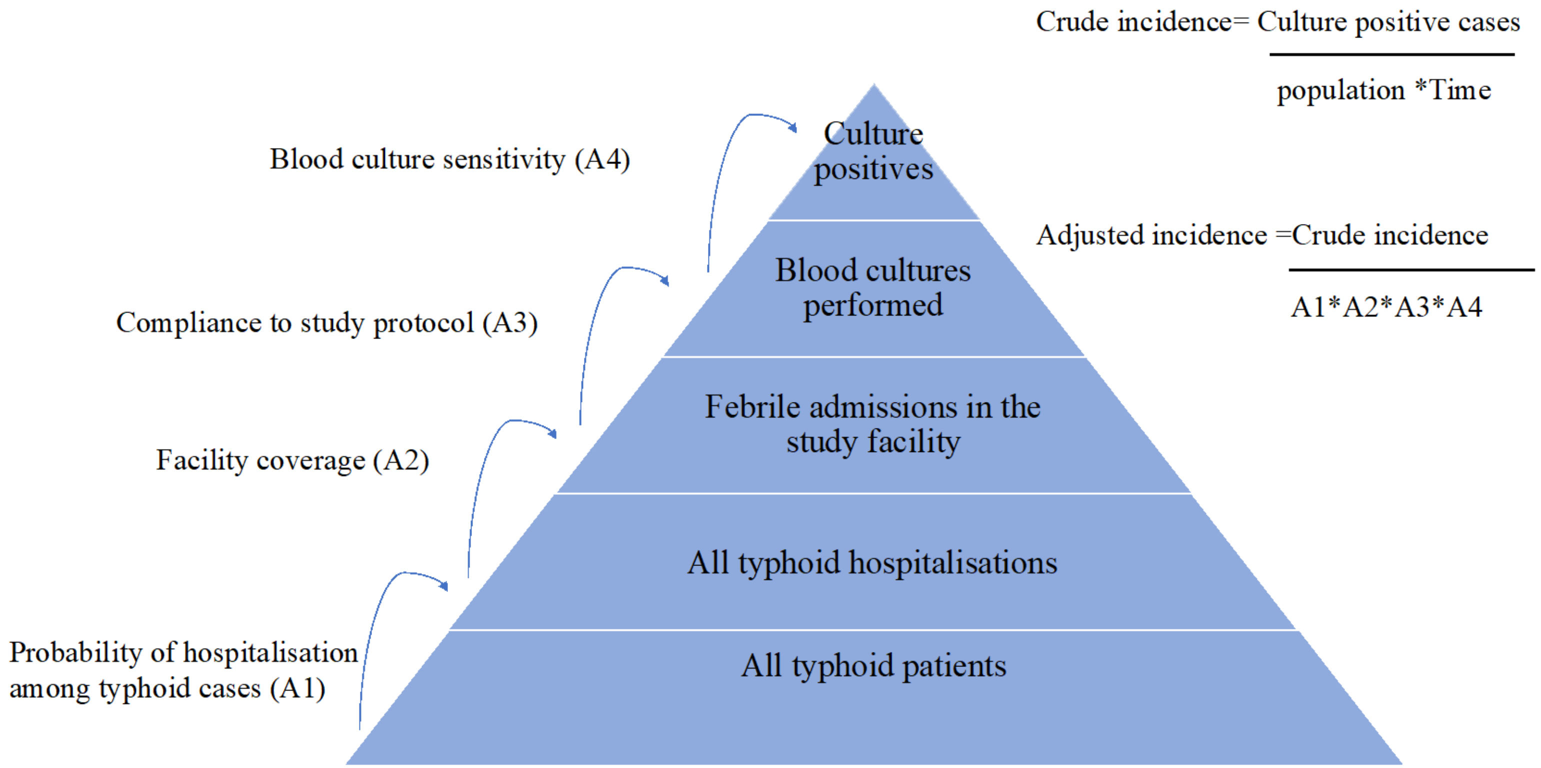

Figure S1: Typhoid disease hybrid surveillance pyramid. The apex represents culture-confirmed typhoid hospitalizations identified in the study hospital. The base of the triangle represents the true burden of typhoid fever in the community. To estimate the true incidence in the community, the crude incidence rate obtained from the hospital-based surveillance is adjusted for A1 (severity of typhoid requiring hospitalization), A2 (proportion seeking healthcare at study facility), A3 (Proportion of hospitalized fevers receiving blood culture), & A4 (sensitivity of blood culture).

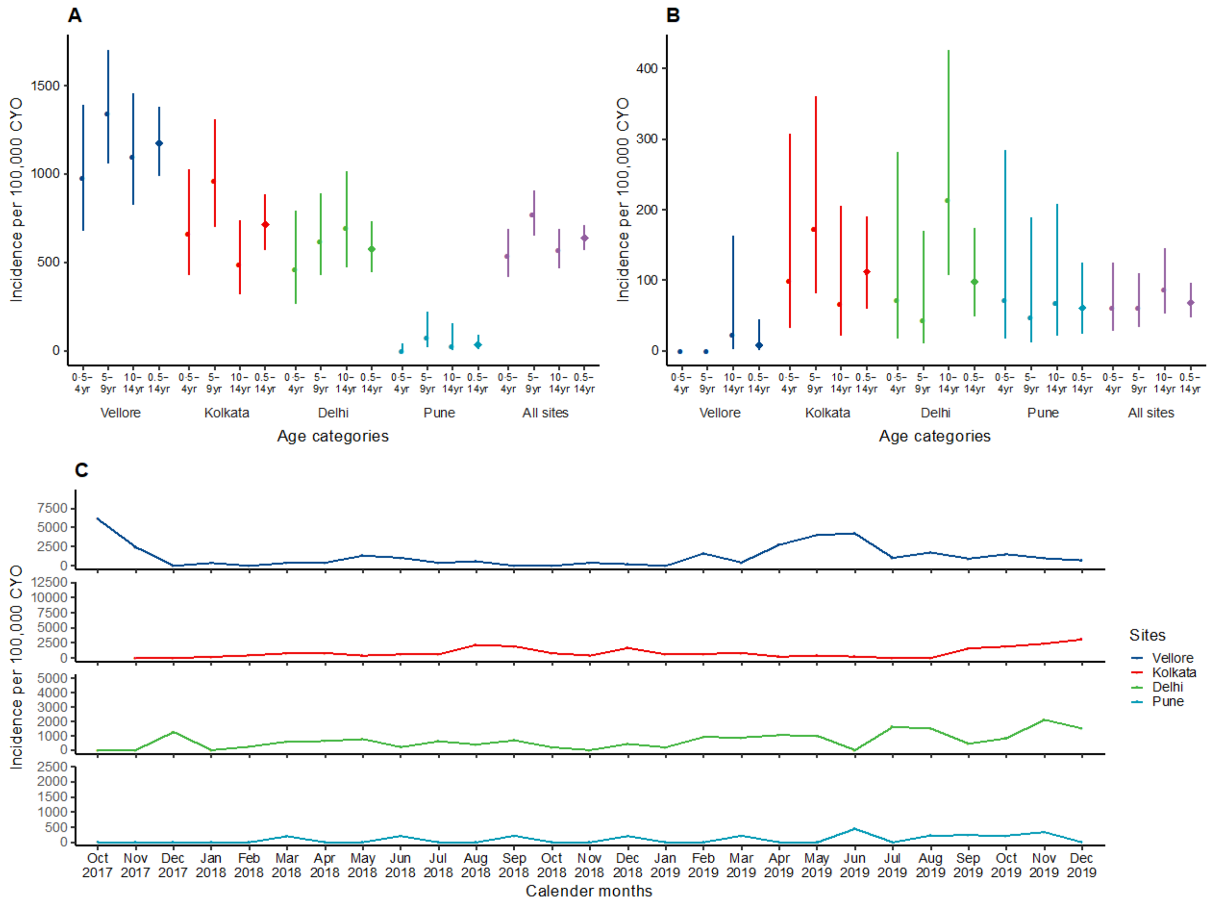

Figure S2: Incidence of culture-confirmed typhoid and paratyphoid fever by Tier 1 cohort site, age, and time.

A) Incidence of typhoid fever and B) incidence of paratyphoid fever by study site and age group. Error bars indicate 95% confidence intervals. C) Monthly incidence (unadjusted) of typhoid and paratyphoid fever at each site with 95% confidence intervals given by the shaded region and periods of high rainfall (monsoon) indicated by the grey rectangles. Incidence in the 0.5-14 years age group is adjusted to match the underlying age-distribution in panel A and B.

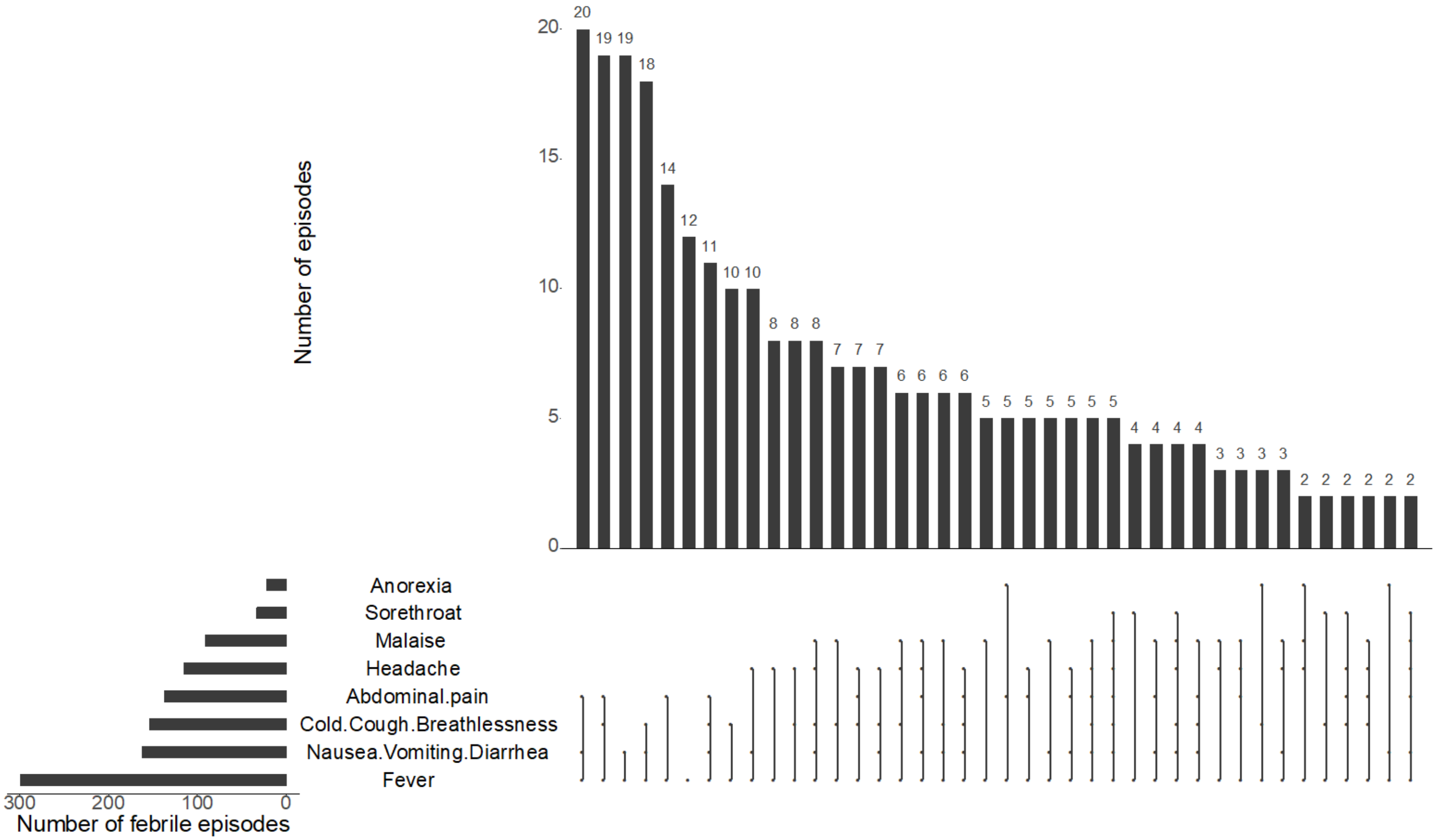

Figure S3: Clinical characteristics of culture-confirmed typhoid cases showing co-occurrence of symptoms, ordered by their frequency across the Tier 1 cohort sites.

Supplementary Tables

Table S1: Cohort study setting and baseline household characteristics for tier 1 surveillance

Site	Vellore	Kolkata	Delhi	Pune	Overall
Type of setting	urban	urban	urban	rural	-
Study area (km^2)	2.2	4.15	0.42	79	
Total number children enrolled	6041	6017	6000	6004	24062
Sex					
Male	3079 (51.0)	3085 (51.3)	3067 (51.1)	3230 (53.8)	12 461 (51.8)
Female	2962 (49.0)	2932 (48.7)	2933 (48.9)	2774 (46.2)	11 601 (48.2)
Age at enrolment					
0.5-4 years	2466 (40.8)	2017 (33.5)	1926 (32.1)	2000 (33.3)	8409 (34.9)
5-9 years	2320 (38.4)	2000 (33.2)	2547 (42.5)	2004 (33.4)	8871 (36.9)
10-14 years	1255 (20.8)	2000 (33.2)	1527 (25.5)	2000 (33.3)	6782 (28.2)
Reported typhoid vaccination*	488 (8.1)	103 (1.7)	62 (1.0)	92 (1.5)	745 (3.1)
Total number of households	3214	4106	3123	3696	14 139
Mean household size (SD)	5.3 (1.9)	4.9 (1.6)	5.3 (1.9)	5.0 (2.0)	5.1 (1.9)
Median monthly family income (IQR) (INR 1000s)	8 (6-10)	9 (8-12)	12 (10-15)	15 (12-25)	10 (8-15)
Separate kitchen available for cooking	2450 (76.2)	1388 (33.8)	2316 (74.2)	2574 (69.6)	8728 (61.7)
Low assets score (< 10)	1490 (46.4)	3032 (73.8)	1333 (42.7)	954 (25.8)	6809 (48.2)
Main source of drinking water					
Piped water into dwelling	577 (18.0)	431 (10.5)	1961 (62.8)	47 (1.3)	3016 (21.3)
Other 'improved' water sources†	2440 (75.9)	3366 (82.0)	815 (26.1)	710 (19.2)	7331 (51.9)
Bottled water	173 (5.4)	96 (2.3)	305 (9.8)	2615 (70.8)	3189 (22.6)
'Unimproved' water sources	24 (0.8)	213 (5.2)	42 (1.3)	324 (8.8)	603 (4.3)
Water treated adequately**	1133 (35.3)	395 (9.6)	1271 (40.7)	915 (24.8)	3714 (26.3)
Type of toilet facility used					
Closed sewer system	0 (..)	1711 (41.7)	40 (1.3)	577 (15.6)	2328 (16.5)
Other sewer system‡	1932 (60.1)	116 (2.8)	14 (0.5)	181 (4.9)	2243 (15.9)
Independent sewer system§	1053 (32.8)	2274 (55.4)	3069 (98.3)	2884 (78.0)	9280 (65.6)
Other unsanitary defecation practices‖	229 (7.1)	5 (0.1)	0 (..)	54 (1.5)	288 (2.0)
Shared toilet facility with other households	536 (16.7)	3181 (77.5)	785 (25.1)	752 (20.4)	5254 (37.2)
Improved sanitation facility available	1061 (33.0)	4094 (99.7)	3118 (99.8)	3634 (98.3)	11 907 (84.2)
Sanitary disposal of child's faeces practised	2411 (75.0)	3757 (91.5)	3026 (96.9)	3397 (91.9)	12 591 (89.1)

Data are n (%) except where indicated. SD=standard deviation. IQR=inter-quartile range. INR=Indian rupees.*Typhoid vaccination reported at the time of screening † include piped water into the yard, public tap/ standpipe, tubewell/borewell, protected well, & springs **Boiling, chlorination, filtration, solar disinfection, and using electronic purifiers were considered as adequate water treatment ‡ include pit latrine, flush/pour flushes to elsewhere. §include septic tank and pit latrine with slab. ||include pit latrine without slab, open pit/composting/hanging toilet, bush/field/no facility

Table S2: Incidence of fever and potential enteric fever across the four cohorts

	Vellore	Kolkata	Delhi	Pune	Overall
Incidence of fever (number, rate per CYO (95% confidence interval))					
0.5-4 years	9505, 3.08 (3.02-3.15)	6175, 2.05 (2.00-2.10)	5612, 1.98 (1.93-2.03)	7503, 2.67 (2.61-2.73)	28 795, 2.45 (2.43-2.48)
5-9 years	8710, 1.72 (1.68-1.75)	6071, 1.49 (1.45-1.53)	5412, 1.15 (1.22-1.18)	7441, 1.76 (1.72-1.80)	27 634, 1.53 (1.51-1.55)
10-14 years	5333, 1.22 (1.19-1.25)	5495, 1.21 (1.18-1.24)	3415, 0.91 (0.88-0.94)	5355, 1.20 (1.16-1.23)	19 598, 1.14 (1.13-1.16)
Total*	23 548, 1.95 (1.92-1.97)	17 741, 1.60 (1.57-1.62)	14 439, 1.41 (1.39-1.43)	20 299, 1.95 (1.92-1.97)	76 027, 1.73 (1.72-1.74)
Median duration of fever, days (IQR)	2 (1- 3)	2 (1- 3)	2 (1- 4)	2 (1- 3)	2 (1- 3)
Incidence of potential enteric fever‡ (number, rate per 100 CYO (95% confidence inter[illegible]					
0.5-4 years	2893, 93.9 (90.5- 97.4)	1671, 55.3 (52.8-58.1)	1592, 56.2 (53.5-59.1)	2032,72.2 (69.2-75.5)	8188, 69.7 (68.3- 71.2)
5-9 years	2580, 50.9 (48.9-52.9)	1483, 36.4 (34.6-38.3)	1634, 34.8 (33.2-36.5)	2138, 50.6 (48.5-52.8)	7835, 43.4 (42.4-44.4)
10-14 years	1405, 32.1 (30.5-33.8)	1131, 24.9 (23.5-26.4)	972, 25.9 (24.3- 27.6)	1380, 30.8 (29.2-32.5)	4888, 28.5 (27.7-29.3)
Total*	6878, 57.1 (55.7-58.4)	4285, 39.4 (38.2-40.5)	4198, 40.6 (39.4- 41.8)	5550, 53.3 (52.0-54.7)	20911, 47.8 (47.2-48.4)
Proportion all fever that were potential enteric fever (PEF) (%)	29	24	29	27	28
Number of PEFs eligible for blood culture$ (%)	4610 (67.0)	2900 (67.7)	3183 (75.8)	3955 (71.3)	14648 (70.1)
Proportion of PEFs receiving antibiotics	4354 (63.3)	2956 (69.0)	2813 (67.4)	4014 (72.3)	14155 (67.7)
Time to initiation of antibiotic from fever onset among PEFs					
First day	419 (9.6)	410 (13.9)	513 (18.1)	1262 (31.4)	2604 (18.4)
Second day	598 (13.7)	501 (17.0)	444 (15.7)	1475 (36.8)	3018 (21.3)
Third day	560 (12.9)	353 (11.9)	476 (16.8)	771 (19.2)	2160 (15.3)
Fourth day	1758 (40.4)	1190 (40.3)	782 (27.6)	329 (8.2)	4059 (28.7)
Fifth day	606 (13.9)	356 (12.0)	309 (10.9)	109 (2.7)	1380 (9.9)
Six or more days	413 (9.5)	146 (4.9)	307 (10.8)	68 (1.7)	934 (6.6)
Number of blood cultures performed among eligible PEF$^{\Sigma}$ (%)	4156 (90.2)	2293 (79.1)	2533 (79.6)	3701 (93.6)	12683 (86.6)
Blood culture not performed for eligible PEF	454 (9.8)	607 (20.9)	650 (20.4)	254 (6.4)	1965 (13.4)

Reasons for not performing blood culture for eligible PEF					
Refusal	247 (5.4)	160 (5.5)	539 (16.9)	129 (3.2)	1075 (7.3)
Others	207 (4.5)	447 (15.4)	111 (3.5)	125 (3.1)	890 (6.1)
Antibiotics used before blood culture (%)	929 (22.4)	753 (32.8)	835 (33.0)	2398 (64.8)	4915 (38.8)
Duration between antibiotic initiation and blood culture					
Two days	277 (29.8)	144 (19.1)	156 (18.7)	447 (18.6)	1024 (20.8)
Three days	311 (33.5)	258 (34.3)	251 (30.1)	949 (39.6)	1769 (36.0)
Four days	220 (23.7)	235 (31.2)	263 (31.5)	811 (33.8)	1529 (31.1)
Five days	75 (8.1)	78 (10.4)	84 (10.1)	126 (5.3)	363 (7.4)
Six or more days	46 (5.0)	38 (5.1)	81 (9.7)	65 (2.7)	230 (4.7)

Data are n (%) except where indicated. *Adjusted to match the underlying age distribution of the populations. ¶An episode of fever was counted as a fever from day one to the last day with elevated temperature that was followed by three fever-free days ‡A child with a fever episode of three or more consecutive days was categorized as potential enteric fever (PEF). ∑Children with PEF were eligible for blood culture. $A child who satisfied the PEF criteria but was afebrile over the last 12 hours prior to the time of performing the blood culture. CYO=child-years of observation. IQR=inter-quartile range.

Table S3: Final clinical diagnosis for potential enteric fevers in the surveillance for enteric fever in India (SEFI) cohorts

Site	Vellore	Kolkata	Delhi	Pune	Overall
Probable bacterial infection					
Appendicitis	3 (0.04%)	0 (..)	0 (..)	0 (..)	3 (0.02%)
Bacterial infection	14 (0.2%)	19 (0.48%)	1 (0.03%)	0 (..)	34 (0.19%)
Ear infection	31 (0.45%)	18 (0.46%)	1 (0.03%)	11 (0.3%)	61 (0.35%)
Enteric fever*	174 (2.53%)	105 (2.68%)	71 (2.28%)	13 (0.35%)	363 (2.06%)
Lower respiratory tract infection	546 (7.94%)	1,077 (27.46%)	18 (0.58%)	345 (9.32%)	1,986 (11.27%)
Scrub	2 (0.03%)	1 (0.03%)	0 (..)	0 (..)	3 (0.02%)
Skin & soft tissue infection	22 (0.32%)	20 (0.51%)	1 (0.03%)	5 (0.14%)	48 (0.27%)
Tuberculosis	2 (0.03%)	0 (0.00%)	3 (0.1%)	0 (0.00%)	5 (0.03%)
Urinary tract infection	72 (1.05%)	5 (0.13%)	2 (0.06%)	5 (0.14%)	84 (0.48%)
Possible bacterial infection					
Acute gastroenteritis	177 (2.57%)	154 (3.93%)	17 (0.55%)	149 (4.02%)	497 (2.82%)
Eye infections	18 (0.26%)	5 (0.13%)	0 (..)	0 (..)	23 (0.13%)
Fever with rash	79 (1.15%)	6 (0.15%)	0 (..)	0 (..)	85 (0.48%)
Other infections	51 (0.74%)	10 (0.25%)	2 (0.06%)	2 (0.05%)	65 (0.37%)
Pharyngitis	160 (2.33%)	0 (0.00%)	0 (0.00%)	1 (0.03%)	161 (0.91%)
Tonsillitis	163 (2.37%)	45 (1.15%)	6 (0.19%)	10 (0.27%)	224 (1.27%)
Unlikely bacterial infection					
Dengue	129 (1.88%)	10 (0.25%)	0 (..)	0 (..)	139 (0.79%)
Jaundice	43 (0.63%)	7 (0.18%)	2 (0.06%)	1 (0.03%)	53 (0.3%)
Measles	2 (0.03%)	6 (0.15%)	0 (..)	1 (0.03%)	9 (0.05%)
Mumps	36 (0.52%)	7 (0.18%)	0 (..)	26 (0.7%)	69 (0.39%)
Upper respiratory tract infection	3,180 (46.23%)	1,396 (35.59%)	377 (12.11%)	501 (13.53%)	5,454 (30.96%)
Varicella	34 (0.49%)	15 (0.38%)	3 (0.1%)	32 (0.86%)	84 (0.48%)
Acute undifferentiated fever	1,940 (28.22%)	1016 (25.91%)	2,610 (83.82%)	2,601 (70.24%)	8,167 (46.36%)
Total	6,878	3,922	3,114	3,703	17,617

* Enteric fever includes clinical diagnosis without blood culture

Table S4: Factors associated with the risk of typhoid in the final multivariable proportional hazards model

Risk factor	Univariable hazard ratio	p-value	Multivariable hazard ratio	p-value
Rural vs urban study site	0.04 (0.02-0.11)	<0.001	0.05 (0.02-0.15)	<0.001
Age in years	0.98 (0.95-1.00)	0.09	0.98 (0.96-1.01)	0.28
Received typhoid vaccine	0.71 (0.54-1.50)	0.37	0.60 (0.28-1.27)	0.18
Family size less than six	0.68 (0.54-0.86)	0.001	0.71 (0.56-0.90)	0.004
Low asset score (<10)	1.57 (1.24-1.98)	<0.001	1.23 (0.97-1.55)	0.08
Improved sanitary facility	0.37 (0.29-0.47)	<0.001	0.50 (0.39-0.63)	<0.001

Table S5: Clinical characteristics and treatment of typhoid fever cases in the cohorts

	Typhoid n=299	Paratyphoid n=33
Symptoms other than fever		
Diarrhea	58 (19.4)	4 (12.1)
Nausea/vomiting	139 (46.5)	11 (33.3)
Abdominal pain	137 (45.8)	17 (51.5)
Loss of appetite	22 (7.4)	1 (3.0)
Cough	145 (48.5)	17 (51.5)
Headache	115 (38.5)	9 (27.3)
Sore throat	33 (11.0)	3 (9.1)
Body pain	18 (6.0)	0 (..)
Duration of fever (days), median (IQR)	9 (7-11)	8 (7-10)
Highest documented temperature (□F), median (IQR)	102.8 (101.6-103.7)	102.2 (100.9-103.7)
Hospitalized	46 (15.4)	7 (21.2)
**Antibiotics taken**	296 (99.0)	33(100)
Azithromycin	230 (76.9)	18 (54.6)
Cephalosporin	145 (48.5)	25 (75.8)
Penicillin	47 (15.7)	8 (24.2)
Others	92 (30.8)	13 (39.4)
Monotherapy	136 (45.9)	13 (39.4)
Multiple	160 (54.1)	20 (60.6)
**Proportion susceptible to different antibiotics**[*]	n tested = 294	n tested=33
Ampicillin	293[†] (99.7)	33 (100)
Chloramphenicol	293[†] (99.7	32 (97.0)
Co-trimoxazole	293 (99.7	32 (97.0)
Ceftriaxone	294 (100)	33 (100)
Azithromycin	288 (98.0)	25 (75.8)
Ciprofloxacin	5 (1.7)	1 (3.0)

[*]Antibiotic susceptibility testing results were not available for 4 children; [†]same isolate was non-susceptible to chloramphenicol and co-trimoxazole

Table S6: Recruitment details and socio-demographic profile of patients from the Tier 2 hospital-based surveillance

		Chandigarh	Anantapur	East Champaran	Nandurbar	Karimganj	Kullu	Overall
**Recruitment details- n**	Total admissions	20632	36629	24016	6812	26631	7353	122073
	Total AFI admissions	5036	4917	2030	2244	4264	1531	20022
	Total AFI patients enrolled	1650 (32.8)	4508 (91.7)	1,743 (85.9)	2234 (99.5)	4249 (99.6)	1352 (88.3)	15736 (78.6)
	Total blood cultures done n (%)	1600 (97.0)	2884 (64.0)	1592(91.3)	2208 (98.8)	3653 (86.0)	1327 (98.2)	13264 (84.3)
**Among all enrolled (% positive)**	*S.* Typhi	92 (5.8)	53 (1.8)	16 (1.0)	12 (0.5)	25 (0.7)	23 (1.7)	221 (1.7)
	S. Paratyphi	41 (2.6)	5 (0.2)	4 (0.2)	1 (0.1)	2 (0.1)	2 (0.1)	55 (0.4)
**From catchment area (% positive)**	*S.* Typhi	92 (5.8)	27 (0.9)	16 (1.0)	9 (0.4)	19 (0.5)	22 (1.7)	185 (1.4)
	S. Paratyphi	41 (2.6)	3 (0.1)	4 (0.3)	1 (0)	1 (0)	2 (0.2)	52 (0.4)
**Sex- n (%)**	Male	918 (55.6)	2507 (55.6)	889 (51.0)	1194 (53.5)	2038 (48.0)	707 (52.3)	8253 (52.5)
**Age- n (%)**	6m-5 years	89 (5.4)	1699 (37.7)	233 (13.4)	149 (6. 7)	1024 (24.1)	316 (23.4)	3510 (22.3)
	6-10 years	80 (4.9)	817 (18.1)	97 (5.6)	111 (5.0)	344 (8.1)	127 (9.4)	1576 (10.0)
	11-15 years	104 (6.3)	444 (9.9)	82 (4.7)	123 (5.5)	218 (5.1)	63 (4.7)	1034 (6.6)
	>=15 years	1377 (83.5)	1548 (34.3)	1331 (76.4)	1851 (82.9)	2663 (62.7)	846 (62.6)	9616 (61.1)
**Highest education Mean (SD)**	No of years	10.9 (3.8)	9.3 (4.8)	10.9 (4.7)	9.4 (4.9)	9.3 (3.2)	11.7 (3.9)	9.8 (4.3)
**Highest occupation n (%)**	Student	0 (..)	0 (..)	0 (..)	0 (..)	0 (..)	0 (..)	0 (..)
	Unemployed	4 (0.2)	5 (0.1)	10 (0.6)	3 (0.1)	3 (0.1)	3 (0.2)	28 (0.2)
	Daily wage	371 (22.5)	4054 (89.9)	410 (23.5)	1893 (84.7)	3189 (75.1)	152 (11.2)	10069 (64.0)
	Salaried	961 (58.2)	423 (9.4)	621 (35.6)	266 (11.9)	762 (17.9)	327 (24.2)	3360 (21.4)
	Business	304 (18.4)	24 (0.5)	387 (22.2)	72 (3.2)	294 (6.9)	870 (64.6)	1951 (12.4)
	Household work	10 (0.6)	2 (0.04)	315 (18.1)	0 (..)	1 (0.02)	0 (..)	328 (2.1)
**Type of house- n (%)**	Hut	4 (0.2)	1524 (33.8)	18 (1.0)	1 (0.04)	99 (2.3)	1 (0.1)	1647 (10.5)
	Kutcha	30 (1.8)	611 (13.6)	598 (34.3)	1776 (79.5)	2682 (63.1)	338 (25.0)	6035 (38.4)
	Pucca	1596 (96.7)	2370 (52.6)	1012 (58.1)	441 (19.7)	991 (23.3)	766 (56.7)	7176 (45.6)
	Mansion	0 (..)	0 (..)	20 (1.2)	0 (..)	3 (0.1)	82 (6.1)	105 (0.7)
	Mixed	20 (1.2)	3 (0.1)	95 (5.5)	16 (0.7)	474 (11.2)	165 (12.2)	773 (4.9)

Table S7: Incidence of hospitalized typhoid fever per 100,000 person years of observation from the Tier 2 hospital-based surveillance

Site	Age group	Person years	Culture confirmed	Crude incidence*	Proportion severe (95% range) (A1)	Study facility utilization (95% range) (A2)	Compliance to the protocol (A3)	Blood culture Sensitivity (95% range) (A4)	Adjusted incidence *	95% Uncertainty Interval #
**Chandigarh**	Paediatric	71595	21	29.3	0.15 (0.12, 0.20)	0.23 (0.12, 0.39)	0.89	0.6 (0.5, 0.7)	1622.4	858.2 – 3358.8
	Adult	193571	71	36.7	0.15 (0.12, 0.20)	0.42 (0.35, 0.50)	0.98	0.6 (0.5, 0.7)	969.9	683 – 1420
	Overall	265164	92	34.7	0.15 (0.12, 0.20)	0.38 (0.32, 0.45)	0.97	0.6 (0.5, 0.7)	1024	723.2 – 1493
**Anantapur**	Paediatric	194244	19	9.8	0.15 (0.12, 0.20)	0.23 (0.13, 0.36)	0.65	0.6 (0.5, 0.7)	730.2	419.4 – 1356.2
	Adult	776975	8	1.0	0.15 (0.12, 0.20)	0.16 (0.10, 0.22)	0.62	0.6 (0.5, 0.7)	119.3	72.7 - 201.9
	Overall	971220	27	2.8	0.15 (0.12, 0.20)	0.18 (0.13, 0.24)	0.64	0.6 (0.5, 0.7)	273.8	178.2 - 433.1
**East Champaran**	Paediatric	392098	1	0.3	0.15 (0.12, 0.20)	0.36 (0.23, 0.51)	0.66	0.6 (0.5, 0.7)	11.9	7.4 - 20.5
	Adult	667627	15	2.3	0.15 (0.12, 0.20)	0.19 (0.13, 0.28)	0.99	0.6 (0.5, 0.7)	130.3	80.8 - 217.8
	Overall	1059725	16	1.5	0.15 (0.12, 0.20)	0.24 (0.18, 0.31)	0.91	0.6 (0.5, 0.7)	76.5	50.8 - 118.7
**Nandurbar**	Paediatric	129095	2	1.6	0.15 (0.12, 0.20)	0.13 (0.04, 0.27)	0.98	0.6 (0.5, 0.7)	151.4	60.9 - 415.3
	Adult	485642	7	1.4	0.15 (0.12, 0.20)	0.09 (0.06, 0.15)	0.99	0.6 (0.5, 0.7)	173.5	97.7 - 317.1
	Overall	614737	9	1.5	0.15 (0.12, 0.20)	0.10 (0.06, 0.15)	0.99	0.6 (0.5, 0.7)	168.6	99.7 - 292.7
**Karimganj**	Paediatric	260043	6	2.3	0.15 (0.12, 0.20)	0.63 (0.38, 0.84)	0.83	0.6 (0.5, 0.7)	48.1	31.2 - 87.8
	Adult	504789	13	2.6	0.15 (0.12, 0.20)	0.30 (0.2, 0.41)	0.88	0.6 (0.5, 0.7)	108.4	69.3 - 177.4
	Overall	764834	19	2.5	0.15 (0.12, 0.20)	0.35 (0.26, 0.46)	0.86	0.6 (0.5, 0.7)	90	60.2 - 140
**Kullu**	Paediatric	51211	5	9.8	0.15 (0.12, 0.20)	0.37 (0.14, 0.66)	0.96	0.6 (0.5, 0.7)	307.8	154.7 - 815.1
	Adult	192650	17	8.8	0.15 (0.12, 0.20)	0.36 (0.23, 0.50)	0.99	0.6 (0.5, 0.7)	274.9	171.9 - 468.9
	Overall	243860	22	9.0	0.15 (0.12, 0.20)	0.36 (0.24, 0.49)	0.98	0.6 (0.5, 0.7)	283	181.6 - 464.7
**All sites**	Paediatric	1098286	54	4.9	0.15 (0.12, 0.20)	0.29 (0.23, 0.35)	0.76	0.6 (0.5, 0.7)	250.5	173.1 - 373
	Adult	2821254	131	4.6	0.15 (0.12, 0.20)	0.24 (0.21, 0.27)	0.90	0.6 (0.5, 0.7)	239	172.1 - 341.5
	Overall	3919540	185	4.7	0.15 (0.12, 0.20)	0.25 (0.22, 0.28)	0.84	0.6 (0.5, 0.7)	247.3	179.5 - 351.1

* per 100,000 Person Years. # Estimated using Monte Carlo simulation

The 95% range for the A1 and A4 adjustment factors were sampled from beta distributions with shape parameters (A1: 46, 253) and (A4: 57, 38), respectively, based on certainty of these estimates. The 95% range for the A2 adjustment factor was sampled from a normal distribution with a logistic transformation.

Table S8: Incidence of hospitalized paratyphoid fever per 100,000 person years of observation from the Tier 2 hospital-based surveillance

Site	Age group	Person years	Culture confirmed	Crude incidence*	Proportion severe (95% range) (A1)	Study facility utilization (95% range) (A2)	Compliance to the protocol (A3)	Blood culture Sensitivity (95% range) (A4)	Adjusted incidence*	95% Uncertainty Interval #
**Chandigarh**	Paediatric	71595	9	12.6	0.15 (0.12, 0.20)	0.23 (0.12, 0.39)	0.89	0.6 (0.5, 0.7)	696.2	368.3 – 1439.3
	Adult	193571	32	16.5	0.15 (0.12, 0.20)	0.42 (0.35, 0.50)	0.98	0.6 (0.5, 0.7)	437.1	308 - 640.5
	Overall	265164	41	15.5	0.15 (0.12, 0.20)	0.38 (0.32, 0.45)	0.97	0.6 (0.5, 0.7)	456.3	322.3 - 665.7
**Anantapur**	Paediatric	194244	3	1.5	0.15 (0.12, 0.20)	0.23 (0.13, 0.36)	0.65	0.6 (0.5, 0.7)	115.3	66.3 - 213.7
	Adult	776975	0	0	0.15 (0.12, 0.20)	0.16 (0.10, 0.22)	0.62	0.6 (0.5, 0.7)	0	-
	Overall	971220	3	0.3	0.15 (0.12, 0.20)	0.18 (0.13, 0.24)	0.64	0.6 (0.5, 0.7)	30.4	19.8 - 48.1
**East Champaran**	Paediatric	392098	0	0	0.15 (0.12, 0.20)	0.36 (0.23, 0.51)	0.66	0.6 (0.5, 0.7)	0	-
	Adult	667627	4	0.6	0.15 (0.12, 0.20)	0.19 (0.13, 0.28)	0.99	0.6 (0.5, 0.7)	34.8	21.5 - 58.1
	Overall	1059725	4	0.4	0.15 (0.12, 0.20)	0.24 (0.18, 0.31)	0.91	0.6 (0.5, 0.7)	19.1	12.7 - 29.7
**Nandurbar**	Paediatric	129095	0	0	0.15 (0.12, 0.20)	0.13 (0.04, 0.27)	0.98	0.6 (0.5, 0.7)	0	-
	Adult	485642	1	0.2	0.15 (0.12, 0.20)	0.09 (0.06, 0.15)	0.99	0.6 (0.5, 0.7)	24.8	14 - 45.3
	Overall	614737	1	0.2	0.15 (0.12, 0.20)	0.10 (0.06, 0.15)	0.99	0.6 (0.5, 0.7)	18.7	11.1 - 32.6
**Karimganj**	Paediatric	260043	0	0	0.15 (0.12, 0.20)	0.63 (0.38, 0.84)	0.83	0.6 (0.5, 0.7)	0	-
	Adult	504789	1	0.2	0.15 (0.12, 0.20)	0.30 (0.2, 0.41)	0.88	0.6 (0.5, 0.7)	8.3	5.3 - 13.6
	Overall	764834	1	0.1	0.15 (0.12, 0.20)	0.35 (0.26, 0.46)	0.86	0.6 (0.5, 0.7)	4.7	3.2 - 7.4
**Kullu**	Paediatric	51211	0	0	0.15 (0.12, 0.20)	0.37 (0.14, 0.66)	0.96	0.6 (0.5, 0.7)	0	-
	Adult	192650	2	1.0	0.15 (0.12, 0.20)	0.36 (0.23, 0.50)	0.99	0.6 (0.5, 0.7)	32.4	20.2 - 55.2
	Overall	243860	2	0.8	0.15 (0.12, 0.20)	0.36 (0.24, 0.49)	0.98	0.6 (0.5, 0.7)	25.7	16.5 - 42.3
**All sites**	Paediatric	1098286	12	1.1	0.15 (0.12, 0.20)	0.29 (0.23, 0.35)	0.76	0.6 (0.5, 0.7)	55.7	38.5 - 82.7
	Adult	2821254	40	1.4	0.15 (0.12, 0.20)	0.24 (0.21, 0.27)	0.90	0.6 (0.5, 0.7)	73	52.5 - 104.4
	Overall	3919540	52	1.3	0.15 (0.12, 0.20)	0.25 (0.22, 0.28)	0.84	0.6 (0.5, 0.7)	69.5	50.5 - 98.6

* per 100,000 Person Years. # Estimated using Monte Carlo simulation

The 95% range for the A1 and A4 adjustment factors were sampled from beta distributions with shape parameters (A1: 46, 253) and (A4: 57, 38), respectively, based on certainty of these estimates. The 95% range for the A2 adjustment factor was sampled from a normal distribution with a logistic transformation.

Table S9: Sensitivity analysis on blood culture sensitivity with preceding antibiotics usage on incidence of typhoid fever

Site	Age group	Culture confirmed	Blood culture sensitivity (95% range)	Adjusted incidence*	95% Uncertainty Interval #
**Chandigarh**	Paediatric	21	0.56 (0.47, 0.66)	1728.2	911 - 3578.2
	Adult	71	0.58 (0.48, 0.67)	1012.2	710.5 - 1486.3
	Overall	92	0.57 (0.47, 0.67)	1071.6	754.1 - 1569.5
**Anantapur**	Paediatric	19	0.59 (0.49, 0.68)	784.4	429.2 - 1387.6
	Adult	8	0.58 (0.48, 0.67)	123.9	75.4 - 210.1
	Overall	27	0.58 (0.48, 0.68)	282	182.8 - 446.7
**East Champaran**	Paediatric	1	0.57 (0.47, 0.67)	12.5	7.7 - 21.6
	Adult	15	0.58 (0.48, 0.67)	136	84.1 - 227.6
	Overall	16	0.57 (0.47, 0.67)	80	53 - 124.4
**Nandurbar**	Paediatric	2	0.6 (0.5, 0.7)	151.4	61 - 417.8
	Adult	7	0.6 (0.5, 0.69)	174.3	98 - 318
	Overall	9	0.6 (0.5, 0.69)	169.5	99.9 - 294.5
**Karimganj**	Paediatric	6	0.59 (0.49, 0.69)	48.5	31.5 - 88.8
	Adult	13	0.58 (0.48, 0.68)	111.3	71 - 182.3
	Overall	19	0.59 (0.49, 0.68)	92	61.3 - 143.1
**Kullu**	Paediatric	5	0.57 (0.47, 0.66)	325.7	163.2 - 861.2
	Adult	17	0.57 (0.47, 0.66)	290.6	180.8 - 496.8
	Overall	22	0.57 (0.47, 0.66)	299.1	191.1 - 493.6
**All sites**	Paediatric	54	0.59 (0.49, 0.68)	256.8	177.3 - 382.9
	Adult	131	0.58 (0.48, 0.68)	246.3	177.1 - 352.9
	Overall	185	0.58 (0.48, 0.68)	254.4	184.3 - 362.2

* per 100,000 Person Years. # Estimated using Monte Carlo simulation

This sensitivity analysis used data on the proportion of participants with antibiotics prior to blood culture collection by age group and site, incorporating a lower sensitivity estimate (40%) for blood cultures taken from these study participants.

Table S10: Sensitivity analysis on blood culture sensitivity with preceding antibiotics usage on incidence of paratyphoid fever

Site	Age group	Culture confirmed	Blood culture sensitivity (95% UI) #	Adjusted incidence*	95% Uncertainty Interval #
**Chandigarh**	Paediatric	9	0.56 (0.47, 0.66)	740.5	391.1 - 1538.4
	Adult	32	0.58 (0.48, 0.67)	456.1	320.5 - 670
	Overall	41	0.57 (0.47, 0.67)	477.4	336 - 699.3
**Anantapur**	Paediatric	3	0.59 (0.49, 0.68)	118.2	67.7 - 219.4
	Adult	0	0.58 (0.49, 0.68)	0	-
	Overall	3	0.58 (0.48, 0.68)	31.3	20.3 - 49.6
**East Champaran**	Paediatric	0	0.57 (0.47, 0.67)	0	-
	Adult	4	0.58 (0.48, 0.67)	36.2	22.4 - 60.6
	Overall	4	0.57 (0.47, 0.67)	20	13.2 - 31.1
**Nandurbar**	Paediatric	0	0.6 (0.5, 0.7)	0	-
	Adult	1	0.6 (0.5, 0.69)	24.9	14 - 45.5
	Overall	1	0.6 (0.5, 0.69)	18.8	11.1 - 32.7
**Karimganj**	Paediatric	0	0.59 (0.49, 0.69)	0	-
	Adult	1	0.58 (0.49, 0.68)	8.6	5.5 - 14
	Overall	1	0.59 (0.49, 0.68)	4.8	3.2 - 7.5
**Kullu**	Paediatric	0	0.57 (0.47, 0.66)	0	-
	Adult	2	0.57 (0.47, 0.66)	34.2	21.3 - 58.5
	Overall	2	0.57 (0.47, 0.66)	27.2	17.4 - 44.8
**All sites**	Paediatric	12	0.59 (0.49, 0.68)	57.1	39.4 - 85.1
	Adult	40	0.58 (0.48, 0.68)	75.2	54.1 - 107.8
	Overall	52	0.58 (0.48, 0.68)	71.5	51.8 - 101.8

* per 100,000 Person Years. # Estimated using Monte Carlo simulation

This sensitivity analysis used data on the proportion of participants with antibiotics prior to blood culture collection by age group and site, incorporating a lower sensitivity estimate (40%) for blood cultures taken from these study participants.

Supplementary Methods References

1. Andersen PK, Gill RD. Cox's regression model for counting processes: a large sample study. Ann Statist 1982;10:1100-20.

2. Raju R, Kezia Angelin J, Karthikeyan AS, et al. Healthcare Utilization Survey in the Hybrid Model of the Surveillance for Enteric Fever in India (SEFI) Study: Processes, Monitoring, Results, and Challenges. J Infect Dis 2021;224:S529-s39.

3. Antillon M, Saad NJ, Baker S, Pollard AJ, Pitzer VE. The relationship between blood sample volume and diagnostic sensitivity of blood culture for typhoid and paratyphoid fever: a systematic review and meta-analysis. J Infect Dis 2018;218:S255-s67.
